# Circulating immune cell populations related to primary breast cancer, surgical removal, and radiotherapy revealed by flow cytometry analysis

**DOI:** 10.1186/s13058-021-01441-8

**Published:** 2021-06-05

**Authors:** Sarah Cattin, Benoît Fellay, Antonello Calderoni, Alexandre Christinat, Laura Negretti, Maira Biggiogero, Alberto Badellino, Anne-Lise Schneider, Pelagia Tsoutsou, Alessandra Franzetti Pellanda, Curzio Rüegg

**Affiliations:** 1 Pathology, Department of Oncology, Microbiology and Immunology, Faculty of Science and Medicine, University of Fribourg, CH-1700 Fribourg, Switzerland; 2 Central Laboratory, Hôpital Fribourgeois, CH-1700 Fribourg, Switzerland; 3 Centro Oncologico Varini-Calderoni-Christinat, CH-6900 Lugano, Switzerland; 4 Radiation Oncology Department, Clinica Luganese Moncucco, CH-6900 Lugano, Switzerland; 5 Clinical Research unit, Clinica Luganese Moncucco, CH-6900 Lugano, Switzerland; 6 Breast and Oncology Center, Hôpital Neuchatelois, CH-2300 La Chaux-de-Fonds, Switzerland; 7 Present Address: Service de Radio-Oncologie, Hôpitaux Universitaires de Genève, CH-1205 Geneva, Switzerland

**Keywords:** Breast cancer, Radiotherapy, Granulocytes, Monocytes, CD117, FlowJo, Unsupervised analysis, Biomarker

## Abstract

**Background:**

Advanced breast cancer (BC) impact immune cells in the blood but whether such effects may reflect the presence of early BC and its therapeutic management remains elusive.

**Methods:**

To address this question, we used multiparametric flow cytometry to analyze circulating leukocytes in patients with early BC (n = 13) at the time of diagnosis, after surgery, and after adjuvant radiotherapy, compared to healthy individuals. Data were analyzed using a minimally supervised approach based on FlowSOM algorithm and validated manually.

**Results:**

At the time of diagnosis, BC patients have an increased frequency of CD117^+^CD11b^+^ granulocytes, which was significantly reduced after tumor removal. Adjuvant radiotherapy increased the frequency of CD45RO^+^ memory CD4^+^ T cells and CD4^+^ regulatory T cells. FlowSOM algorithm analysis revealed several unanticipated populations, including cells negative for all markers tested, CD11b^+^CD15^low^, CD3^+^CD4^−^CD8^−^, CD3^+^CD4^+^CD8^+^, and CD3^+^CD8^+^CD127^+^CD45RO^+^ cells, associated with BC or radiotherapy.

**Conclusions:**

This study revealed changes in blood leukocytes associated with primary BC, surgical removal, and adjuvant radiotherapy. Specifically, it identified increased levels of CD117^+^ granulocytes, memory, and regulatory CD4^+^ T cells as potential biomarkers of BC and radiotherapy, respectively. Importantly, the study demonstrates the value of unsupervised analysis of complex flow cytometry data to unravel new cell populations of potential clinical relevance.

**Supplementary Information:**

The online version contains supplementary material available at 10.1186/s13058-021-01441-8.

## Background

Breast cancer (BC) is the most frequent cancer and the main cause of cancer-related mortality for women in industrialized countries [1]. Three clinically relevant biological BC subtypes (i.e., estrogens/progesterone receptor positive (ER+/PR^+^), human epidermal growth factor receptor 2 (HER2) amplified, and triple negative) and multiple molecular subtypes (e.g., Luminal A/B, HER2, basal like, normal like) with distinct features and clinical outcomes have been defined and characterized [2–5].

Early detection and surgery in combination with adjuvant treatments tailored on biological and molecular subtypes have improved patients’ survival by about 30% in the past three decades [6]. The goal of adjuvant therapy, including radiotherapy, is the eradication of tumor cells that are disseminated before diagnosis and surgery. Some of these disseminated tumor cells (DTC), however, will escape therapy and later progress to form metastases, which in most patients represent the main cause of cancer-related death. After breast-conserving surgery, radiotherapy reduces the risk of BC recurrence and death. Among women with operable BC, randomized trials have demonstrated equivalent disease-free and overall survival between mastectomy and breast-conserving surgery followed by radiotherapy alone and/or hormonal, anti-HER2, or chemotherapy [7–16].

Mammography is the standard approach for the detection of asymptomatic BC [17]. In spite of its benefits in reducing BC specific mortality, mammography has some important limitations [18]: low specificity and sensitivity; risk of over-diagnosis; risk of inducing BC due to X-ray exposure, particularly in patients with defective DNA repair genes [19]; and not recommended before the age of 50 in spite of the fact that 20–25% of all BCs appear before this age. There is therefore an unmet need for complementary or alternative methods for the detection of asymptomatic, early BC [20–22]. Circulating tumor cells (CTC), cell-free tumor-derived DNA, mRNA and miRNA, proteins, autoantibodies, and metabolites are being explored as candidate blood-based biomarkers for BC detection, diagnosis, or monitoring, but so far none entered routine clinical practice [23–27]. Similarly, there are no effective blood-based biomarkers to actively assess patients’ response to treatment and monitoring disease state after therapy. Also, the most used blood biomarker in clinical practice, CA 15-3, is not specific and sensitive in early breast cancer diagnosis [28].

Tumors, including BC, mobilize and recruit immuno-inflammatory cells to their microenvironment [29–31]. Monocytic and granulocytic cells, mostly immature forms, as well as lymphocytes, contribute to cancer progression by promoting immunosuppression, angiogenesis, cancer cell survival, growth, invasion, and metastasis [32, 33]. We have previously shown that metastatic BC patients have elevated frequencies of TIE2^+^CD11b^+^ and CD117^+^CD11b^+^ leukocytes circulating in the blood, and that circulating CD11b^+^ cells express higher mRNA levels of the M2 polarization markers CD163, ARG1, and IL-10 [34]. Treatment with paclitaxel in combination with bevacizumab decreased the frequency CD117^+^CD11b^+^ leukocytes, IL-10 mRNA levels in CD11b^+^ cells, and IL-10 protein in plasma. We therefore considered that blood circulating leukocytes, or sub-population, thereof, may reflect cancer-relevant immuno-inflammatory events that may be further explored as BC-associated biomarkers.

Here, we analyzed the phenotype of blood leukocytes of patients with early BC at time of diagnosis, after surgery, and after adjuvant radiotherapy (RTX), relative to healthy donors (HD), using flow cytometry, and a minimally supervised analytical approach based on FlowSOM algorithm and manual validation. We identified with both approaches cell populations associated with the presence of a primary BC, tumor removal, and adjuvant radiotherapy. These results indicate that phenotypical analysis of peripheral blood leukocytes, with a minimally supervised analytical approach, may be a clinically-relevant strategy for the identification of cellular biomarkers for BC detection and therapy monitoring.

## Materials and methods

### Patients and clinical study

The study was approved by the Cantonal ethic commission for human research on Humans of Canton Ticino (CE 2967) and extended to Vaud-Fribourg-Neuchâtel, Switzerland. The study includes 13 female patients (Table 1) who were diagnosed with primary, non-metastatic BC (stage T1–4, N0–N1, M0,). All patients underwent conservative surgery and received standard fractionated adjuvant radiotherapy (2 Gy per session, total dose : 50 + 10 Gy). For the analysis at time of primary detection, only 11 patients were included for comparison with 11 age-matched healthy donors (HDs). Blood samples were collected at the following time-points (Fig. 3): after diagnosis was confirmed histologically but before surgery (Sample 0); after surgery the day of radiotherapy start (immediately before first irradiation, Sample 1); at the last day of radiotherapy (6 weeks after starting radiotherapy, Sample 2); and 6–8 weeks after the end of the radiotherapy (for the majority to the patients this was 12 weeks after starting radiotherapy, Sample 3). All Patients and HDs gave written informed consent before study entry. Patients were recruited before surgery at Clinica Luganese Moncucco, Lugano, and at Hôpital Neuchâtelois, La Chaux-de-Fonds, once diagnosis was histologically confirmed. Mean age for cancer patients was 60.6 years (all patients were between 43 and 73 years old). HDs were recruited along the study, based on the following criteria: age-matched relative to BC patients, no regular medications in the last 6 months, no previous cancer diagnosis, no chronic diseases, and normal blood analyses at time of recruitment.

**Table 1:** Clinical-pathological data of breast cancer patients included in the study

Patient number	Age	ER (%)	PR (%)	HER2 (+/−)	Ki67 (%)	Grade	Tumor size	LN mets	Anti-hormonal therapy
1	62	95	70	−	5	1	pT1b	pN1a	−
2	58	95	95	−	10	2	T1b	N0	−
3	50	100	100	+	5	1	pT1	pN0	Tamoxifen
4	73	95	2	−	25	2	pT2	pN1a	−
5	49	90	80	−	10	2	T1b	pN0	Tamoxifen
6	69	100	100	−	15	2	pT1a	pN0	Tamoxifen
7	53	95	60	−	10	2	pT1c	pN0	Letrozole
8	73	100	0	−	20	na	pT1c	pN0	Tamoxifen
9	67	90	80	−	5	1	pT1b	pN0	Tamoxifen
10	66	100	100	−	5	2	pT1c	pN0	Letrozole
11	64	95	80	−	10	1	pT1b	pN0	Letrozole
12	61	80	100	−	10	2	pT1b	pN0	Anastrozole
13	43	95	95	−	10	2	pT1c	pN0	Tamoxifen

Patient’s demographics, tumor subtype, grade, stage (pT and pN), and anti-hormonal treatment after conservative surgery. ER (%), estrogen receptor expression in percent; PR (%), progesterone receptor expression in percent; HER2 (±), overexpression of HER-2; Ki67 (%), fraction of cancer cells positive for Ki67 expression

### Blood processing

Twenty milliliters of peripheral venous blood was collected using BD Vacutainer® Blood Collection EDTA Tubes (Becton Dickinson, Franklin Lakes, NJ, USA) following the manufacturer’s instructions and immediately shipped by courier at room temperature to the laboratory. All analyses were performed within 24 h after blood collection. Antibody staining was performed in whole blood. Plasma and total leukocytes were isolated from the remaining blood using BD Vacutainer® CPT™ Cell Preparation Tube (Becton Dickinson) with sodium heparin following the manufacturer’s instructions. Plasma fraction was frozen at – 80 °C and isolated leukocytes were lysed in RA1 lysis buffer (Macherey-Nagel, Düren, Deutschland) and stored at – 80 °C.

### Flow cytometry

Whole blood stainings were performed within 24 h after blood collection. Leukocytes were counted using Cell-Dyn Sapphire Hematology System (Abbott Diagnostics, Chicago, IL, USA). For staining, 1 million cells per tube were used based on direct blood cell count. Directly labeled antibodies were added to whole blood and incubated for 20 min at 4 °C, followed by 10 min red-blood-cells lysis (Bühlmann Laboratories, Schönenbuch, Switzerland) and subsequently washed using cold PBS. All anti-human antibodies were used at the concentrations recommended by the manufacturer: anti-CD15-PeCy7 (clone HI98), anti-CD14-Pe (clone MφP9), anti-CD163-FITC (clone GHI/61), anti-CD11b-BV510 (clone ICRF44), anti-CD33-V450 (clone WM53), anti-CD64-APCH7 (clone 10.1), anti-CD117-APC (clone YB5.B8), anti-CD45RA-PeCy7 (clone HI100), anti-CD25-Pe (clone M-A251), anti-CD4-FITC (clone RPA-T4), anti-CD8-V500 (clone SK1), anti-CD45RO-BV421 (clone UCHL1), anti-CD3-APCH7 (clone SK7), and CD127-Alexa Fluor 647 (clone HIL-7R-M21) and 7AAD (all from Becton Dickinson). BD FACSCanto II (Becton Dickinson) instrument was used to analyze samples and FlowJo 10.6.2 (Treestar Inc., Ashland, OR, USA) software and several software plugins (FlowCLEAN, downsample_V3, FlowSOM, tSNE) were used to analyze all data.

### Reverse transcription real-time PCR (RT-qPCR)

Total mRNA from total white blood cells was extracted using the NucleoSpin RNA kit from Macherey-Nagel following the manufacturer’s instructions (Düren, Germany). The purity and quantity of all RNA samples were examined by NanoDrop (Witec AG, Luzern, Switzerland). Total RNA was retro-transcribed using M-MLV reverse transcriptase kit following the manufacturer’s instructions (ThermoFisher Scientific, Waltham, Massachusetts, USA) using 500 ng of total RNA. cDNA was subjected to amplification by real-time qPCR with the StepOne SYBR System (Life Technologies) using the following primer pairs (Eurofins Genomics, Huntsville, AL, USA) at the indicated hybridization temperatures: GAPDH 58 °C (Fw-TCTTCTTTTGCGTCGCCAGC, Rev-GATTTTGGAGGGATCTCGCTCCT), ARG1 58 °C (Fw-GGAGTCATCTGGGTGGATGC, Rev-CTGGCACATCGGGAATCTTTC), IL-10 58 °C (Fw-CGAGATGCCTTCAGCAGAGT, Rev-AATCGATGACAGCGCCGTAG), CD117 57 °C (Fw-GATTATCCCAAGTCTGAGAATGAA, Rev-CGTCAGAATTGGACACTAGGA), FN1 52 °C (Fw- ACTTCGACAGGACCACTTGA, Rev-TCAAATTGGAGATTCATGGGA). Real-time PCR data were then analyzed using the comparative Ct method [35].

### Statistical analysis

Acquired data were analyzed and graphics were generated using Prism Software (GraphPad, La Jolla, CA, USA). Samples with incomplete staining due to technical problems during the antibody staining process or during acquisition were excluded from the statistical analysis. Statistical comparisons between cancer patients and healthy donors were performed by T test assuming non-homogenous variance. Normality distribution of the samples was checked in case of significance and, if non-Gaussian, a Mann-Whitney replaced the T test results. Statistical comparisons of all time-points to observe the effect of radiotherapy were performed by one-way ANOVA assuming non-homogenous variance using Tukey correction. Normality distribution of the samples was checked in case of significance and, if non-Gaussian, a Kruskal-Wallis assay replaced the ANOVA results. Results were considered to be significant from p < 0.05. In the figures, the various p values thresholds are presented as follows: ≤ 0.05 = *, ≤ 0.01 = **, ≤ 0.001 = ***, ≤ 0.0001 = ****.

## Results

### Increased frequency of CD117^+^ CD11b^+^ granulocytes in the peripheral blood of patients with newly diagnosed non-metastatic BC

Based on previous observations made in metastatic BC patients [34], we hypothesized that an increased frequency of circulating CD11b^+^ cells expressing CD117 and/or displaying a M2 activation phenotype may also occur in patients with early BC. To test this hypothesis, we monitored the frequency of leukocyte populations in the blood of 11 non-metastatic BC patients (cT1–4, N0–1, M0) (Table 1) at time of diagnosis using flow cytometry. Aged-matched women without BC served as the control population (healthy donors (HDs)). Monocytes were defined as CD11b^+^CD33^+^CD14^high^CD15^−^ and granulocytes as CD11b^+^CD33^+^CD14^low^CD15^+^ cells (Supplementary Figure S1). Due to limitations at acquisition, we could not add additional markers to further characterize these cell populations. In both cell populations, we monitored the expression of CD117 (cKit), the receptor for Kit-ligand/stem cell factor widely present in hematopoietic progenitor cells [36, 37], and CD163, a M2 polarization marker in monocytes [38]. In order to avoid investigator-associated biases and variability in the results inherent to supervised manual analysis of flow cytometry data, we develop a minimally supervised, standardized analytical workflow based on the FlowSOM algorithm (Supplementary Figure S2), in complement to conventional manual gating and supervised analysis.

**Fig. 1:**
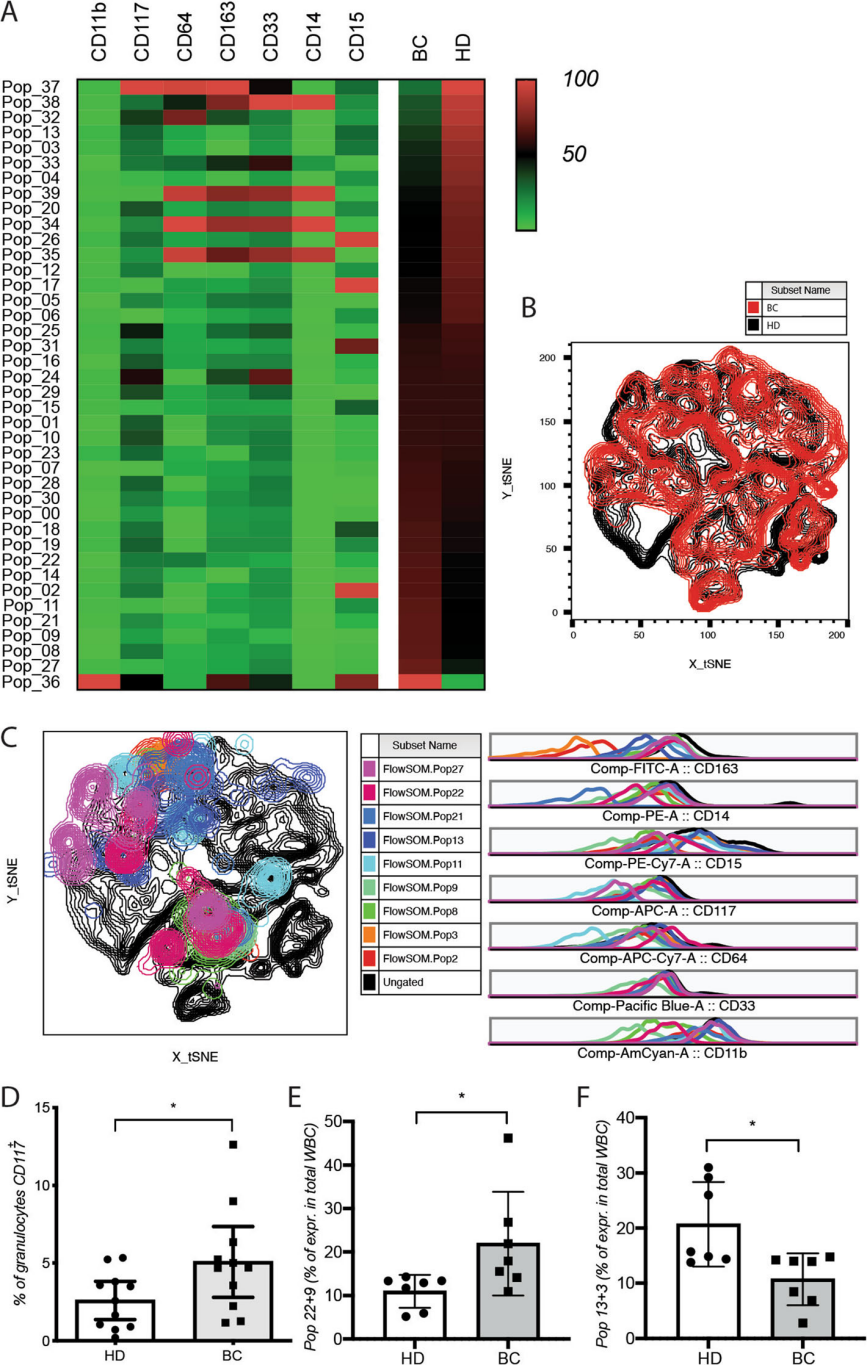
Altered frequency of circulating monocytic populations in cancer patients. **A** Heat map of the FlowSOM clustering between breast cancer patients (BC) and healthy donors (HD). tSNE visualization of **B** the monocytic expression profile and **C** the differentially expressed clusters in the blood of breast cancer patients at the time of the first diagnosis vs healthy donors. Frequency of **D** CD117^+^ granulocytic population, and the atypical populations **E** 22 + 9 and **F** 13 + 3 at the same timing. WBC, white blood cells. Cell analysis and quantification were performed by flow cytometry with FlowJo software and results are represented as mean values +/− SD

Cells clusters revealed by standardized analytical workflow were considered of interest when their frequency was more than 10% different between HDs and cancer patients. Some of the 14 analyzed clusters corresponded to non-standard populations, for example, those negative for all tested markers or expressing unanticipated marker combinations (Fig. 1A–C). These populations would have been missed by conventional supervised gating and analysis driven by the marker combination of interest. Interestingly, we observed a significant increase in the frequency of CD117^+^ cells among the circulating granulocytic population in cancer patients relative to HDs (Fig. 1D). Patients over 61 years of age, but not HDs, had a non-significant trend toward having more circulating CD117^+^ cells compared to younger patients (less than 55 years old) (not shown).

We observed a similar (but non-significant) trend in the frequency of monocytic cells, albeit at lower frequency. In addition, the frequencies of some non-classical cell populations, such as those expressing none of the markers of interest (Cluster 13 and 3) or a CD11b^+^CD15^low^ cell population (Cluster 22+9), were significantly different between BC patients and HDs (Fig. 1E, F). No significant changes were observed for CD163^+^ cells in both granulocytes and monocytes cell populations (Supplementary Figure S3). No effect related to the age of the patients or HDs was observed in this small cohort (not shown).

### Non-standard CD3-expressing cells are present with increased frequency in the peripheral blood of newly diagnosed BC patients

In parallel, we monitored the presence of selected lymphocyte populations in both groups. By conventional supervised analysis, we observed no differences in classical CD3^+^CD4^+^ T cells, CD3^+^CD8^+^ T cells, and CD3^+^CD4^+^CD25^+^CD127^−^ regulatory T cells (Tregs). Likewise, we observed no changes in the frequency of memory (CD45RO^+^CD45RA^−^) or naïve (CD45RA^+^CD45RO^−^) T cells within the same lymphocyte populations (Supplementary Figure S4).

**Fig. 2:**
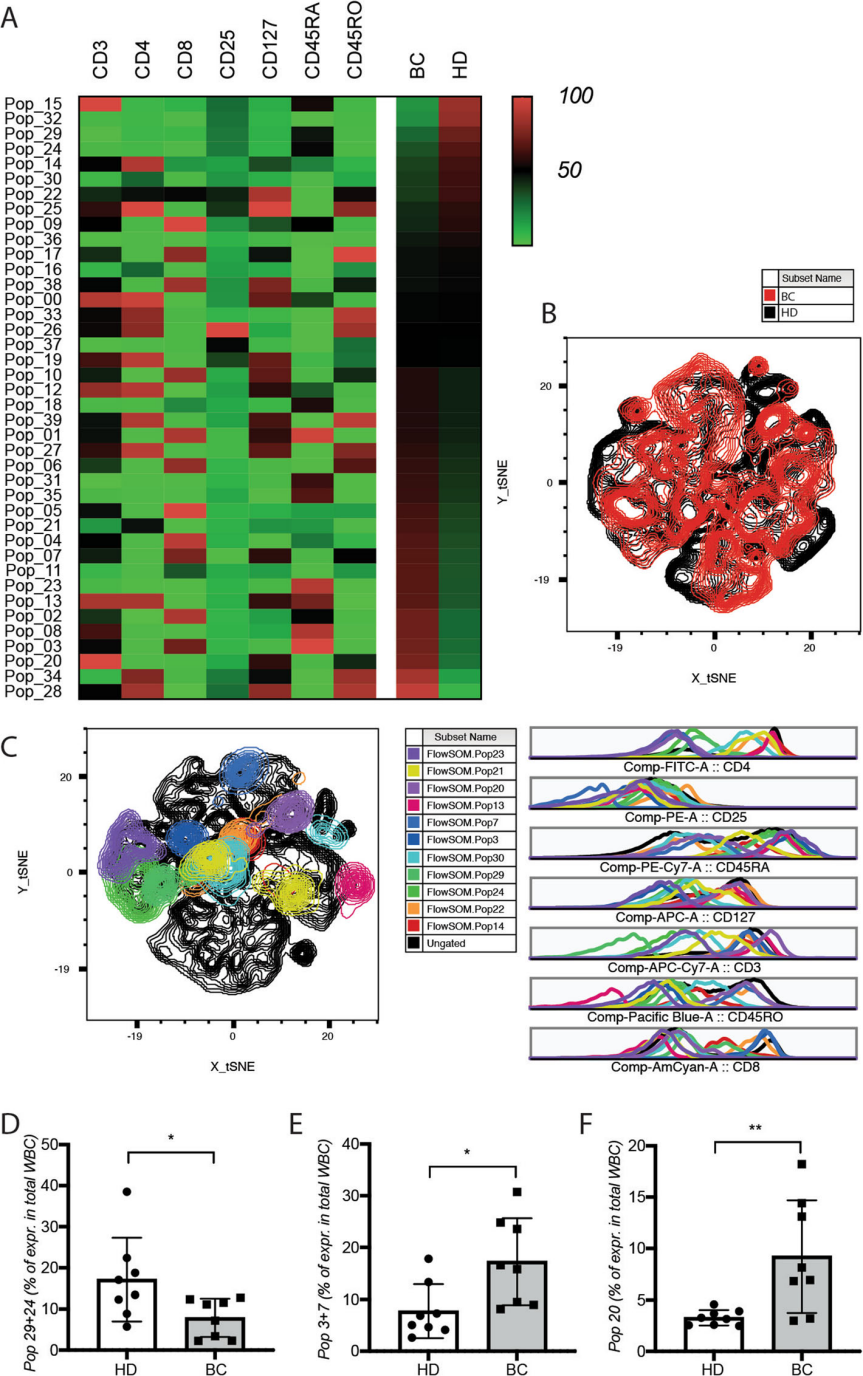
Altered frequency of circulating lymphocyte populations in cancer patients. **A** Heat map of the FlowSOM clustering between breast cancer patients (BC) and healthy donors (HD). tSNE visualization of **B** the lymphocyte expression profile and **C** the differentially expressed clusters in the blood breast cancer patients at the time of the first diagnosis of healthy donors. Frequency of the atypical populations **D** 29 + 24, **E** 3 + 7, and **F** 20 at the same timing. WBC, white blood cells. Cell analysis and quantification were performed by flow cytometry with FlowJo software and results are represented as mean values +/− SD

In contrast, FlowSOM analysis performed on lymphocytes revealed 21 populations that were more than 10% differentially represented between HDs and cancer patients (Fig. 2A–C). A population of cells of the size of lymphocytes, but negative for CD3, CD4, or CD8 expression (Cluster 24 + 29), was significantly less represented in cancer patients relative to HDs. CD3^+^CD8^+^ T cells expressing CD127 and CD45RO markers (Cluster 3 + 7) are present at a significantly higher frequency in cancer patients. A cell cluster expressing CD3, but not CD4 or CD8 (Cluster 20), was observed at higher frequency in cancer patients relative to HDs (Fig. 2D–F).

Taken together, these results reveal an increased frequency of peripheral blood CD117^+^ granulocytes in non-metastatic BC patients at time of diagnosis, as well as significant changes in the frequency of myeloid and lymphocytic cell populations expressing unconventional marker combinations. They also demonstrate that FlowSOM-based analysis can identify cell populations that would have been missed by supervised analysis.

### No detectable changes in the expression level of transcripts for M2 polarization markers

We previously reported that transcripts of M2-associated genes were expressed at higher levels circulating CD11b^+^ cells in metastatic BC patients compared to HDs (34). We therefore analyzed the expression of mRNA for CD117 and the M2 markers IL-10, fibronectin-1 (FN1), and arginase 1 (ARG1) in total leukocytes from cancer patients and HDs. No differences in expression levels were observed (Supplementary Figure S5).

### A proof-of-concept study to monitor the effects of surgical tumor removal and adjuvant radiotherapy on circulating immune cells in BC patients

**Fig. 3:**
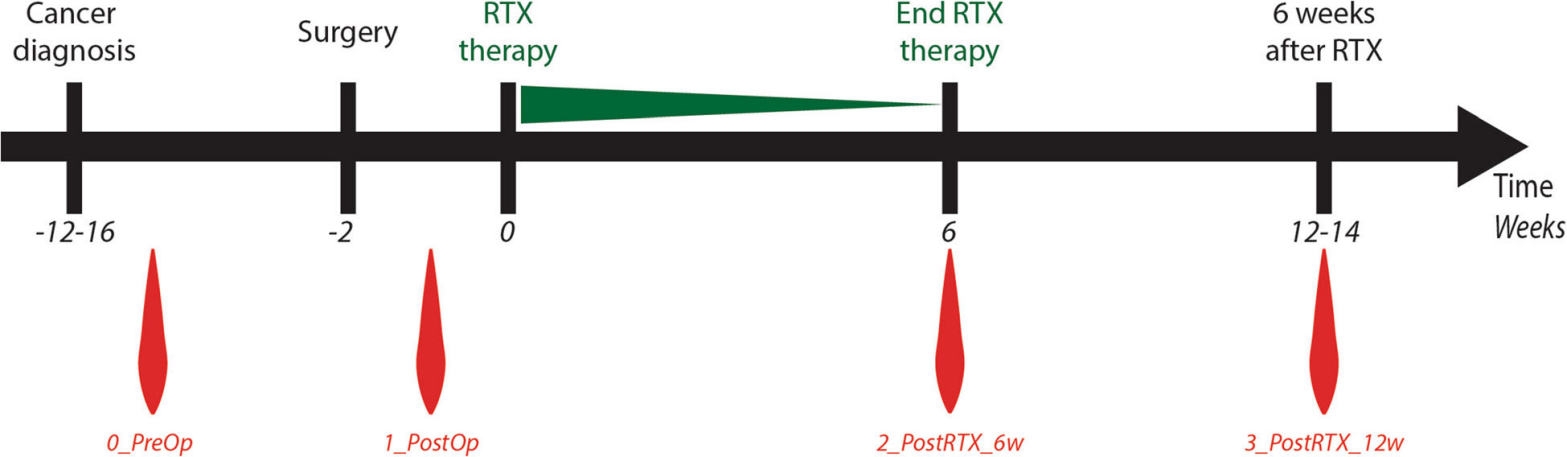
Schematic representation of the radiotherapy study. Patients were enrolled after a confirmed histological diagnosis of breast cancer. All patients underwent conservative surgery and received standard fractionated adjuvant radiotherapy (2 Gy per session, total 50 + 10 Gy). Blood samples were collected after diagnosis was confirmed histologically, but before surgery (Sample 0), after surgery; the day of starting radiotherapy (immediately before fist irradiation, Sample 1); at the last day of radiotherapy (6 weeks after starting radiotherapy, Sample 2), and 6-8 weeks after the end of the radiotherapy (for the majority to the patients this was 12 weeks after starting radiotherapy, Sample 3)

The differences observed in myelomonocytic and lymphocytic populations in BC patients at time of diagnosis relative to HDs raised the question whether tumor removal and/or adjuvant therapy may reverse these changes, or induce additional ones. To address this question, we performed a proof-of-concept study, by taking advantage of the fact that the investigated patients were scheduled for breast conservative tumor removal and adjuvant radiotherapy as part of their standard treatment. Adjuvant radiotherapy was selected as therapy of choice as systemic effects on the immune system have been reported [39–41], while on the other side chemotherapy was excluded in order to avoid that myelosuppressive effects induced by chemotherapy could non-specifically impact the results [42]. To search for potential changes in cell populations in response to surgery and radiotherapy we analyzed monocytes, granulocytes, and lymphocytes at three time-points: after surgery/before radiotherapy start (1_PostOP), at the end of radiotherapy (6 weeks; 2_Post_RTX_6w) and at 6–8 weeks after the end of radiotherapy (12–14 weeks; 3_Post_RTX_12w) in 13 patients. Results were compared to values obtained at the time of diagnosis (0_PreOP) (Fig. 3).

### Tumor removal increased the frequency of monocytic and granulocytic cells, but decreased CD117^+^ granulocytes and radiotherapy-induced changes in non-standard myeloid cell populations

**Fig. 4:**
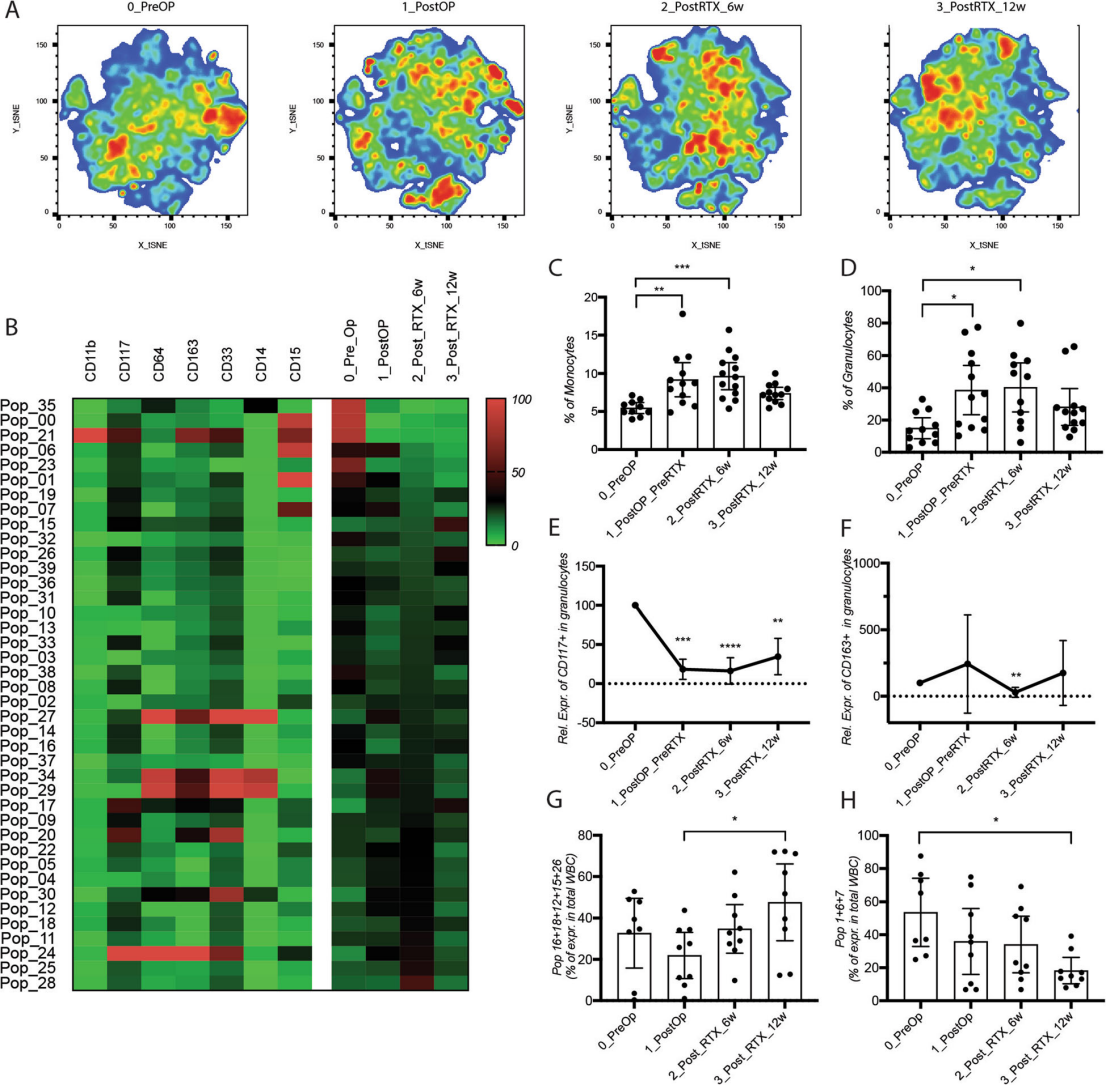
Tumor removal reduces the frequency of circulating CD117^+^ granulocytic cells. **A** Comparative visualization of the expression of surface markers in monocytes at different time-points of treatment by tSNE. **B** Heat map of the FlowSOM clustering of breast cancer patients at indicated time-points. Frequency of **C** monocytes and **D** granulocytes populations in patients at the indicated time-points during treatment. Frequency of **E** CD163^+^ and **F** CD117^+^ granulocyte population at indicated time-points relative to frequency at 0_PreOp time-point. Frequency of the **G** combined 16 + 18 + 21 + 12 + 15 + 26 and **H** 1 + 6 + 7 atypical cell populations during treatment. WBC, white blood cells. Cell analysis and quantification was performed by flow cytometry with FlowJo software and results are represented as mean values +/− SD

Using FlowSOM workflow of analysis, we observed distinct expression profiles at the four time-points globally visualized by tSNE. Seventeen cell clusters were found highly differentially represented in one group compared to the other groups. Surprisingly, when looking at the expression profiles of each cluster of interest, the majority of them was lacking CD33 expression, suggesting that this marker may not be suitable to analyze the monocytes fraction (Fig. 4A, B).

After tumor removal and at the end of radiotherapy the frequency of both monocytes and granulocytes were significantly increased relative to values at the time of diagnosis and returned to pre-therapy levels 6–8 weeks after the end of radiotherapy (Fig. 4C, D). Strikingly, within the granulocytic population, the fractions of CD117 expressing cells significantly decreased after tumor removal and this decrease persisted after the end of radiotherapy (Fig. 4E). Surgery had no impact on the fraction of CD163^+^ granulocytic population (Fig. 4F). Radiotherapy itself had an impact on granulocytes expressing CD163, but not on granulocytes expressing CD117 (Fig. 4E, F).

The presence of one particular cell cluster expressing only CD11b and CD15 (Pop 1, 6, and 7) clearly decreased after radiotherapy. Another population expressing CD11b but lacking expression of all tested markers significantly increased during and after radiotherapy (Pop 16, 18, 12, 15, and 26) (Fig. 4G, H). The latter observation suggests that some populations defined by non-standard marker combinations may be potentially interesting candidates to investigate further with an extended panel of markers.

Analysis of total blood leukocytes for CD117, IL-10, FN1, and ARG1 mRNA expression by RT-qPCR revealed no observable differences in their expression levels (Supplementary Figure S6).

### Adjuvant radiotherapy increased the frequency of CD4^+^ memory and regulatory T cells, and induced changes in non-standard lymphocytic populations

**Fig. 5:**
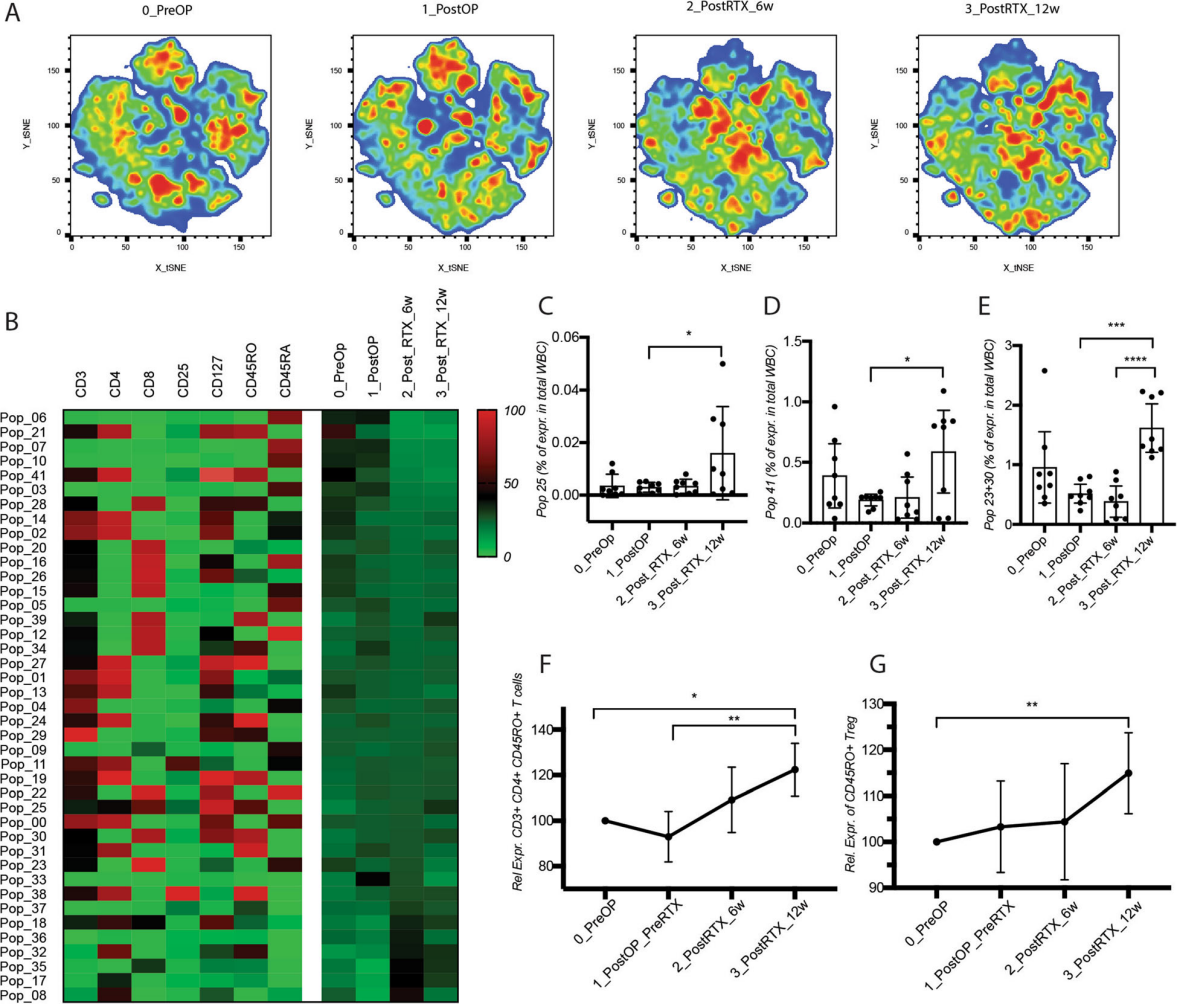
Tumor removal and radiotherapy reduce the fraction of CD117^+^ cells within the granulocytic population. **A** Comparative visualization of the expression of surface markers in lymphocytes at different time-points of treatment by tSNE. **B** Heatmap of the FlowSOM clustering of breast cancer patients at indicated time-points during treatment. Frequency of the atypical populations **C** 25, **D** 41, and **E** 23 + 30 in patients during treatment. Relative quantification to 0_PreOp time-point of **F** CD4+ CD45RO+ lymphocytes and **G** CD45RO+ regulatory T cells during treatment. WBC, white blood cells. Cell analysis and quantification was performed by flow cytometry with FlowJo software and results are represented as mean values +/− SD

Likewise, we performed unsupervised analysis of the lymphocyte populations at the three time-points after surgery and radiotherapy. Visualization by tSNE revealed distinctive changes in marker expression profiles. Eleven cell clusters were found highly differentially represented in one group compared to the other ones (Fig. 5A, B). After tumor removal, we observed highly variable effects on the frequency of T lymphocyte subpopulations, most of which were inconsistent and statistically non-significant.

Among the stably differentially represented clusters at the various time-points, a CD3^+^ cell population positive for CD4 and CD8 (Cluster 25), and a CD3^+^CD4^+^CD127^+^CD45RO^+^ population (Cluster 41) appeared at higher frequency after treatment (Fig. 5C, D). Strikingly, the frequency of this CD45RO^+^RA^−^ memory subset within the CD3^+^CD4^+^ lymphocyte population was significantly and consistently increased at the end of radiotherapy and this increase was still evident 6–8 weeks later. A similar increase was also present among CD4^+^ regulatory T cells, corresponding to clusters 23 and 30, which also persisted after the end of radiotherapy (Fig. 5E–G).

## Discussion

Mammography-based screening significantly reduces BC-related mortality, but intrinsic and practical limitations call for novel screening approaches [17**–**20]. Blood-based biomarkers exploiting CTC, cancer-derived DNA or RNA, are being explored, but so far none reached clinical routine practice [22, 43]. Similarly, there are no validated biomarkers for monitoring patients’ response to treatment or detecting relapses before they become symptomatic.

In this study, we pursued the use of flow cytometry to analyze the phenotype and frequency of blood leukocytes in patients with non-metastatic BC at the time of diagnosis, after surgical tumor removal, and after adjuvant radiotherapy. Using a combination of minimally supervised (FlowSOM algorithm), and supervised (manual) analytical approaches, we report that (i) at diagnosis, BC patients have an increased frequency of circulating CD117^+^ granulocytes relative to age-matched healthy donors; (ii) surgical tumor removal causes a transient increase of monocytes and granulocytes, and a long-lasting decrease of CD117^+^ granulocytes; (iii) radiotherapy significantly increases CD45R0^+^ memory T cells and CD4^+^ Treg cells; and (iv) with the FlowSOM algorithm, we identified additional unanticipated, non-classical cell populations differentially represented between HD and BC patients and in BC patients in response to therapy.

Traditionally, flow cytometry results are analyzed manually, which has the potential to introduce investigator-specific biases. To tested for this, we asked a technician expert with flow cytometry analysis but who was not involved in this project, to reanalyze the raw data and to find four specific populations of interest. Reassuringly this analysis confirmed the original results (not shown). A full reanalysis, however, is time-consuming and hard to implements in a routine (clinical) setting. As an alternative to avoid potential human biases and variability of the result, we choose to perform unsupervised, algorithm-based analyses. Also, algorithms are more effective in finding potentially interesting marker combinations. Populations of interest were then investigated more in detail manually. Here too, we performed several times the same unsupervised analysis and we could reproducibly find the same populations (not shown). This confirms the value of the approach and of the populations identified, further supporting the use of an unsupervised analysis approach to prospectively identify robust changes among complex populations.

One question that emerged during the unsupervised analysis is how to set the cutoff between samples to discriminate robust marker combinations (clusters of interest) form from unstable ones. As no standard or optimal method is defined for this kind of analyses, we tested cut-off values of 5%, 10%, and 20%. The 5% cutoff was not stringent enough, because many identified populations could not be confirmed. With a 10% cutoff, we observed a good balance between identify robust, reproducible populations, and non-interesting or unstable ones. With a 20% cutoff, we missed small but robust differences seen with 5 and 10% cutoff. We therefore decided to use the 10% cutoff.

CD117, the receptor for Kit-ligand/stem cell factor, is widely expressed in hematopoietic progenitor cells in the bone marrow, while CD117^+^ leukocytes are rarely detectable in the circulation under homeostatic conditions [36]. We have previously reported a role of CD117^+^ leukocytes in metastasis in the murine 4T1 metastatic BC model [44] and the presence of CD117^+^CD11b^+^ cells in the blood of mBC patients [34]. Here, we observed an increased frequency of a CD117^+^ population among total granulocytes in the peripheral blood of non-metastatic BC patients at the time of diagnosis, compared to HD. Interestingly, the frequency of CD117^+^ granulocytes significantly dropped upon tumor removal and remained below pre-treatment levels after radiotherapy. Thus, the increased frequency of CD117^+^ granulocytes may reflect the presence of the primary tumor. No changes were observed in CD117 mRNA expression in total leukocytes. This could be due to the fact that CD117^+^ cells are lost during leukocyte isolation for RNA extraction (flow cytometry was performed in non-separated total whole blood), or that CD117 mRNA expression has ceased upon cell mobilization (while CD117 protein persisted at the cell surface). The latter possibility is consistent with our previous observation that mobilized CD117^+^ cells adoptively transferred to a recipient mouse, rapidly became CD117 negative [44]. In contrast, the frequency of CD163^+^ granulocytes remained rather constant, with only a transient decrease during radiotherapy. The implication of this decrease is unclear as CD163 expression did not significantly differ between HD and BC patients at the time of diagnosis. After surgery and radiotherapy also no change in the mRNA expression of CD117 and ARG1, FN1, IL-10 (i.e., M2 polarization markers) was observed, owing probably to the lack of enrichment of CD11b^+^ cells for PCR analysis.

In cancer patients at the time of diagnosis, we observed a higher frequency of atypical T lymphocytes (CD3^+^CD4^−^CD8^−^) and of a population of the size of lymphocytes lacking expression of all the tested markers. These observations suggest that some potentially interesting changes may occur in atypical T cells or in non-T cell populations such as B cells or NK cells. Strikingly, after radiotherapy, we observed a steady and significant increase of the fraction of CD45RO^+^ memory T cells within total CD4^+^ T cells and within CD4^+^ Treg. We also observed the increased presence of a T cell population expressing both CD4 and CD8 markers. This suggests that radiotherapy may cause T cell activation leading to the subsequent generation of memory T cell subsets. All patients included in this study had ER^+^ cancer, and the majority (10/13) received concomitant anti-hormonal treatment. We were thoughtful to the potential effect of anti-hormonal therapy and we analyzed our results by stratifying patients based on anti-hormonal therapy. No significative changes in the cell populations were observed (data not shown). However, as there were only three patients without anti-hormonal treatment, the significance of these results has to be considered with care.

There is increasing evidence that radiotherapy exerts its therapeutic effects, not only in the local treatment field, but also at distant sites (i.e., the so-called abscopal effect), at least in part, by eliciting a T cell immune response [39]. The recent observation that the combination of radiotherapy with immune checkpoint inhibitors in experimental models and cancer patients results in potent synergistic therapeutic effects further supports the involvement of T cell-dependent events and the therapeutic effects of radiotherapy [45**–**48]. Through experimental work and mathematical modeling, it has been proposed that anti-tumor T cells may be mobilized by radiotherapy toward peripheral tissues to eliminate DTC [49**–**51]. However, to date, there is paucity of human data demonstrating specific changes in circulating T lymphocytes to support such a model. Radiotherapy was reported to cause a global reduction in circulating lymphocyte subsets in patients treated for stage I–II prostate cancer [52] or to induce an increase in CD4^+^ Treg in the peripheral blood of patients with diverse solid cancers [53]. Low-dose radon therapy for chronic inflammatory diseases was shown to induce a long-lasting increase in circulating T cells paralleled with a reduced expression of activation markers [54]. Thus, the observed effect of adjuvant radiotherapy on memory CD4^+^ T cells is novel and should be further explored in conjunction with patients’ outcome, as possible biomarkers of therapy response or efficacy.

## Conclusion

Taken together, this human exploratory study in early, non-metastatic BC revealed changes in blood leukocyte populations associated with the presence of BC, surgical removal, and adjuvant radiotherapy. Specifically, we identified CD117^+^ granulocytes and CD45RO^+^ CD4^+^ memory T cells correlating with the presence of the primary tumor and radiotherapy, respectively. Importantly, the study demonstrates that a minimally supervised, algorithm-based analysis of flow cytometry data is a powerful tool to reproducibly detect phenotypical changes in peripheral blood leukocytes in cancer patients. The approach also identifies non-anticipated population correlated with disease state of therapy. These results should instigate the further investigation of peripheral blood leukocytes as a source of reliable candidate biomarkers to detect BC, to monitor response to treatment and possibly disease progression.

## Supplementary Information

**Additional file 1: Figure S1.** Gating strategy for manual flow cytometry. Example of the manual gating strategy applied on flow cytometry data for (A) Monocytes and Granulocytes, (B) Lymphocytes. **Figure S2.** Workflow for unsupervised analysis of flow cytometry data. **Figure S3.** No changes are observed among CD117^+^ monocytes and CD163 expressing monocytic cells in the blood of breast cancer patients vs healthy donors. **Figure S4.** No differences are observed for lymphocytes in the blood of breast cancer patients vs healthy donors. **Figure S5.** No significant differences observed in mRNA levels of CD117 and M2 polarization markers in blood leukocytes of breast cancer patients vs healthy donors. **Figure S6.** No significant differences are observed in mRNA level of CD117 and M2 polarization markers in blood leukocytes of patients during radiotherapy.

## Data Availability

The datasets used and/or analyzed during the current study are available from the corresponding author on reasonable request.

## Abbreviations

BC: Breast cancer
CTC: Circulating tumor cells
DTC: Disseminated tumor cells
HD: Healthy donor
Treg: Regulatory T cell
WBC: White blood cells
