# Supplementary material for: Circulating immune cell populations related to primary breast cancer, surgical removal, and radiotherapy revealed by flow cytometry analysis
Source: Breast Cancer Res. 2021 Jun 5;23:64. doi: 10.1186/s13058-021-01441-8 (PMC8180078; doi:10.1186/s13058-021-01441-8)
Supplement: Supplementary file 1 — Additional file 1: Figure S1. Gating strategy for manual flow cytometry. Example of the manual gating strategy applied on flow cytometry data for (A) Monocytes and Granulocytes, (B) Lymphocytes. Figure S2. Workflow for unsupervised analysis of flow cytometry data. Figure S3. No changes are observed among CD117+ monocytes and CD163 expressing monocytic cells in the blood of breast cancer patients vs healthy donors. Figure S4. No differences are observed for lymphocytes in the blood of breast cancer patients vs healthy donors. Figure S5. No significant differences observed in mRNA levels of CD117 and M2 polarization markers in blood leukocytes of breast cancer patients vs healthy donors. Figure S6. No significant differences are observed in mRNA level of CD117 and M2 polarization markers in blood leukocytes of patients during radiotherapy. [file 13058_2021_1441_MOESM1_ESM.docx]

Supplementary Methods.

Workflow for flow cytometry unsupervised analysis

Manual analysis of complex flow cytometry data sets is time-consuming and the results can be affected by the experience of the person performing the analysis and the expected results [1,2]. In order to standardize and optimize flow cytometry data analysis, and avoid investigator-associated biases, we established a new analytical workflow (See also Supplementary figure S1).

The initial preparation of clean data files to feed the algorithm was still performed partially manually. These manual steps consisted in the automatic compensation of each file, the removal of debris and dead cells based on viability dye, and the exclusion of doublets based on forward-scatter area versus high. The file is then cleaned from any event recorded under conditions of unstable flow using the FlowCLEAN algorithm of FlowJO software [3]. Based on forward and side scattering data, all cell populations or populations of interest, as in our case, are selected for further analysis. Following this initial data cleaning, samples of interest are down-sampled using the plugin in FlowJO to normalize the number of cells between analyzed samples. Then all data are concatenated in one single file in order to analyze and compare all patients together. Finally the FlowSOM algorithm is applied to the concatenated file for unsupervised detection of clusters representing distinct cell populations [4]. Each cluster identified by the FlowSOM algorithm is then compared for its presence or absence in cancer patients and healthy donors, and differentially present cell clusters are investigated and manually to validate the specific expression profile and distribution.

This new analysis workflow allows for a minimally supervised, robust and faster approach to analyze large and complex sets of flow cytometry data while reducing potentially investigator-based biases. This workflow can be applied to high number of patients avoiding time consuming manual gating and is more reproducible then the classical fully manual gating and analysis.

**References to supplementary methods**

1. on behalf of the EuroFlow Consortium (EU-FP6, LSHB-CT-2006-018708); Kalina, T.; Flores-Montero, J.; van der Velden, V.H.J.; Martin-Ayuso, M.; Böttcher, S.; Ritgen, M.; Almeida, J.; Lhermitte, L.; Asnafi, V.; et al. EuroFlow standardization of flow cytometer instrument settings and immunophenotyping protocols. *Leukemia* **2012**, *26*, 1986–2010, doi:10.1038/leu.2012.122.

2. Laskowski, T.J.; Hazen, A.L.; Collazo, R.S.; Haviland, D. Rigor and Reproducibility of Cytometry Practices for Immuno‐Oncology: A multifaceted challenge. *Cytometry* **2020**, *97*, 116–125, doi:10.1002/cyto.a.23882.

3. Fletez-Brant, K.; Špidlen, J.; Brinkman, R.R.; Roederer, M.; Chattopadhyay, P.K. flowClean: Automated identification and removal of fluorescence anomalies in flow cytometry data: flowClean for Quality Control of Flow Cytometry Data. *Cytometry* **2016**, *89*, 461–471, doi:10.1002/cyto.a.22837.

4. Van Gassen, S.; Callebaut, B.; Van Helden, M.J.; Lambrecht, B.N.; Demeester, P.; Dhaene, T.; Saeys, Y. FlowSOM: Using self-organizing maps for visualization and interpretation of cytometry data: FlowSOM. *Cytometry* **2015**, *87*, 636–645, doi:10.1002/cyto.a.22625.

A

B

Supplementary Figure S1. Gating strategy for manual flow cytometry.

Example of the manual gating strategy applied on flow cytometry data for **(A)** Monocytes and Granulocytes, **(B)** Lymphocytes.


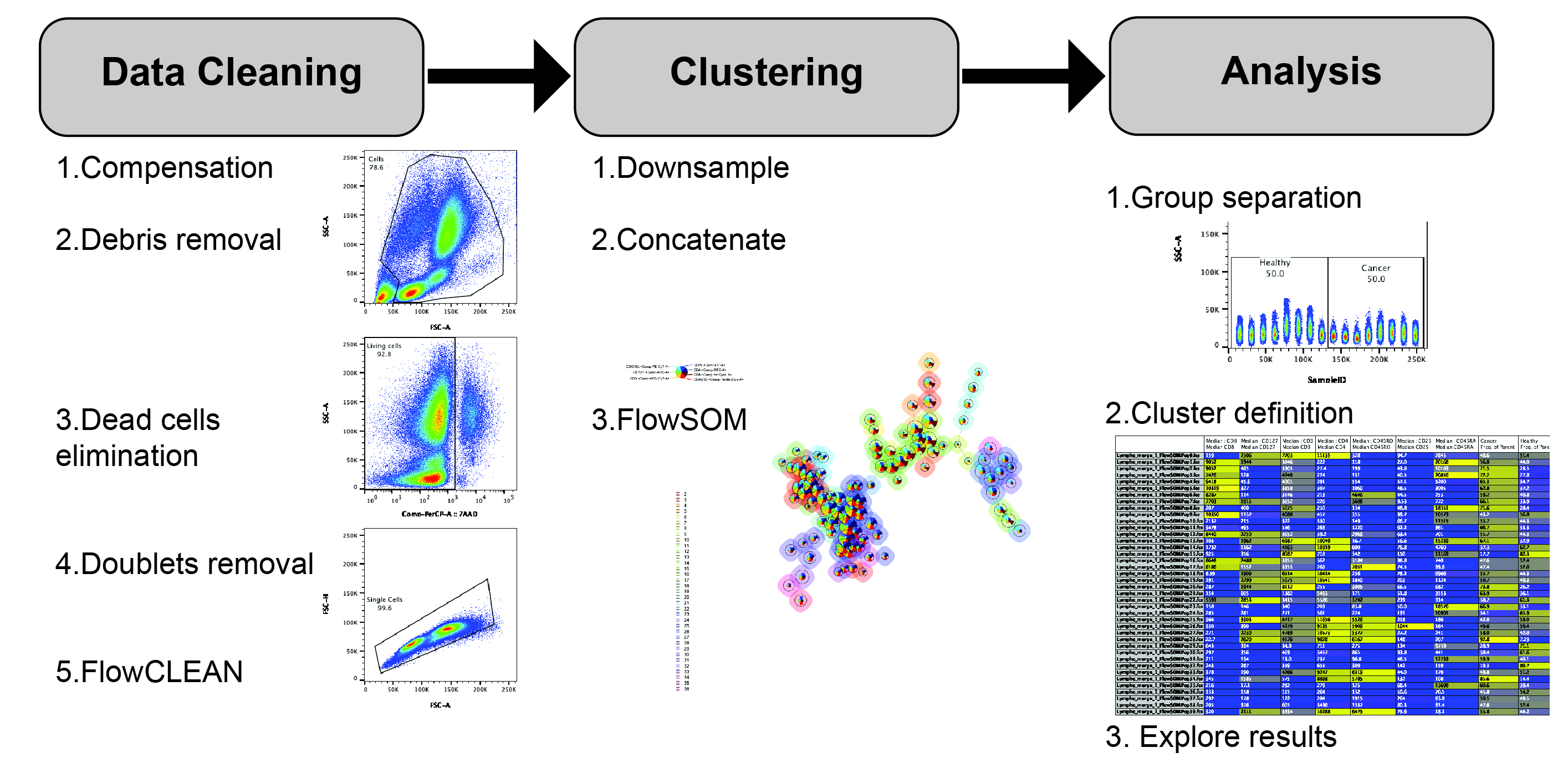


Supplementary Figure S2. Workflow for unsupervised analysis of flow cytometry data.

The analytical process consists of three different steps (data cleaning; data clustering and data analysis) applied to the flow cytometry raw data for reproducible analysis in time, avoid investigator-associated bias and identify unanticipated cell populations.


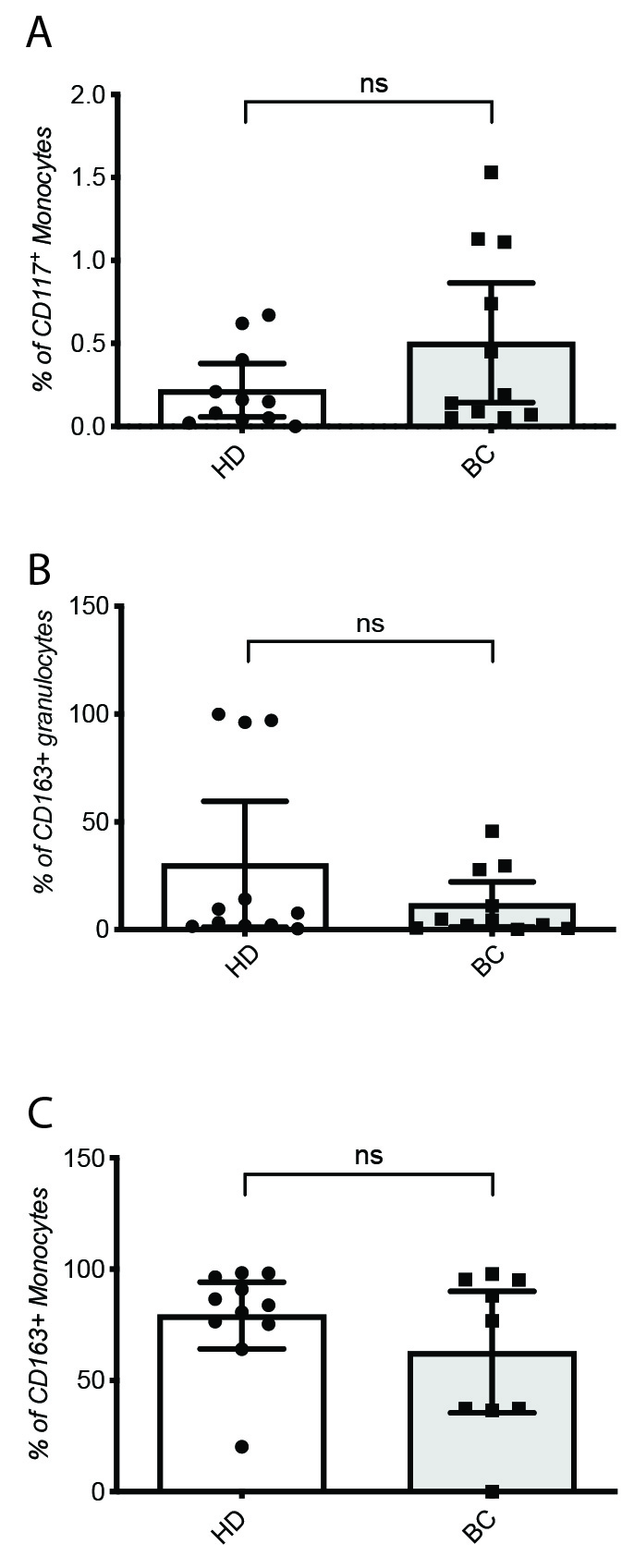


Supplementary Figure S3. No changes are observed among CD117^+^ monocytes and CD163 expressing monocytic cells in the blood of breast cancer patients vs healthy donors.

Frequency of **(A)** CD117^+^ monocytes, **(B)** CD163^+^ granulocytes and **(C)** CD163^+^ monocytes in the blood of healthy donors (HD) and breast cancer patients (BC) at time of first diagnosis. Cell analysis and quantification were performed by flow cytometry with FlowJo software and results are represented as mean values +/- SD. Ns, nonsignificant.


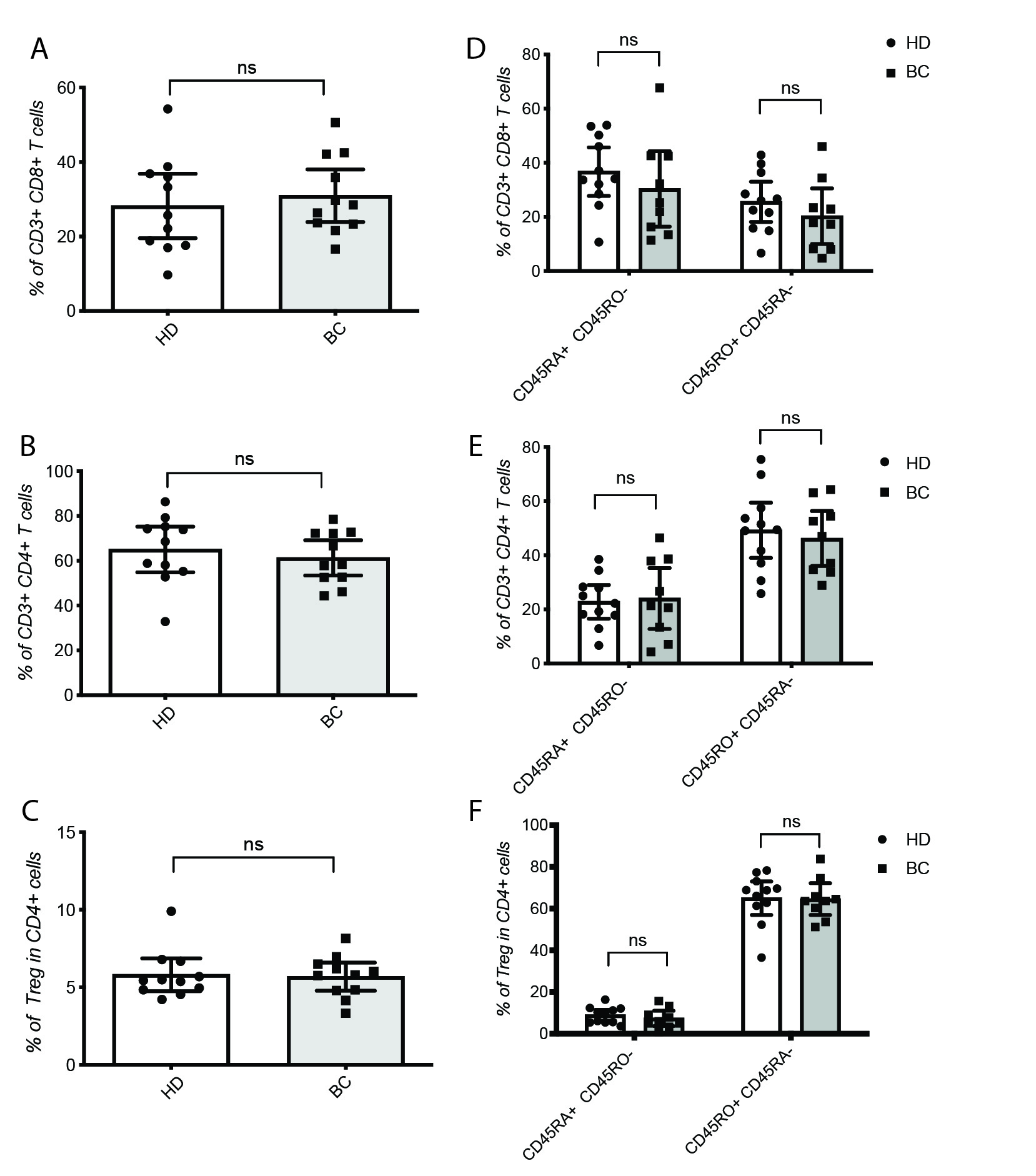


Supplementary Figure S4. No differences are observed for lymphocytes in the blood of breast cancer patients vs healthy donors.

Frequency of **(A)** CD8^+^, **(B)** CD4^+^ T lymphocytes and **(C)** CD4^+^ regulatory T lymphocytes in the blood of healthy donors (HD) and breast cancer patients (BC) at time of first diagnosis. Frequency of naïve and memory phenotypes of **(D)** CD8^+^, **(E)** CD4^+^ T lymphocytes and **(F)** CD4^+^ regulatory T lymphocytes at the same conditions. Cell analysis and quantification were performed by flow cytometry with FlowJo software and results are represented as mean values +/- SD. Ns, nonsignificant.


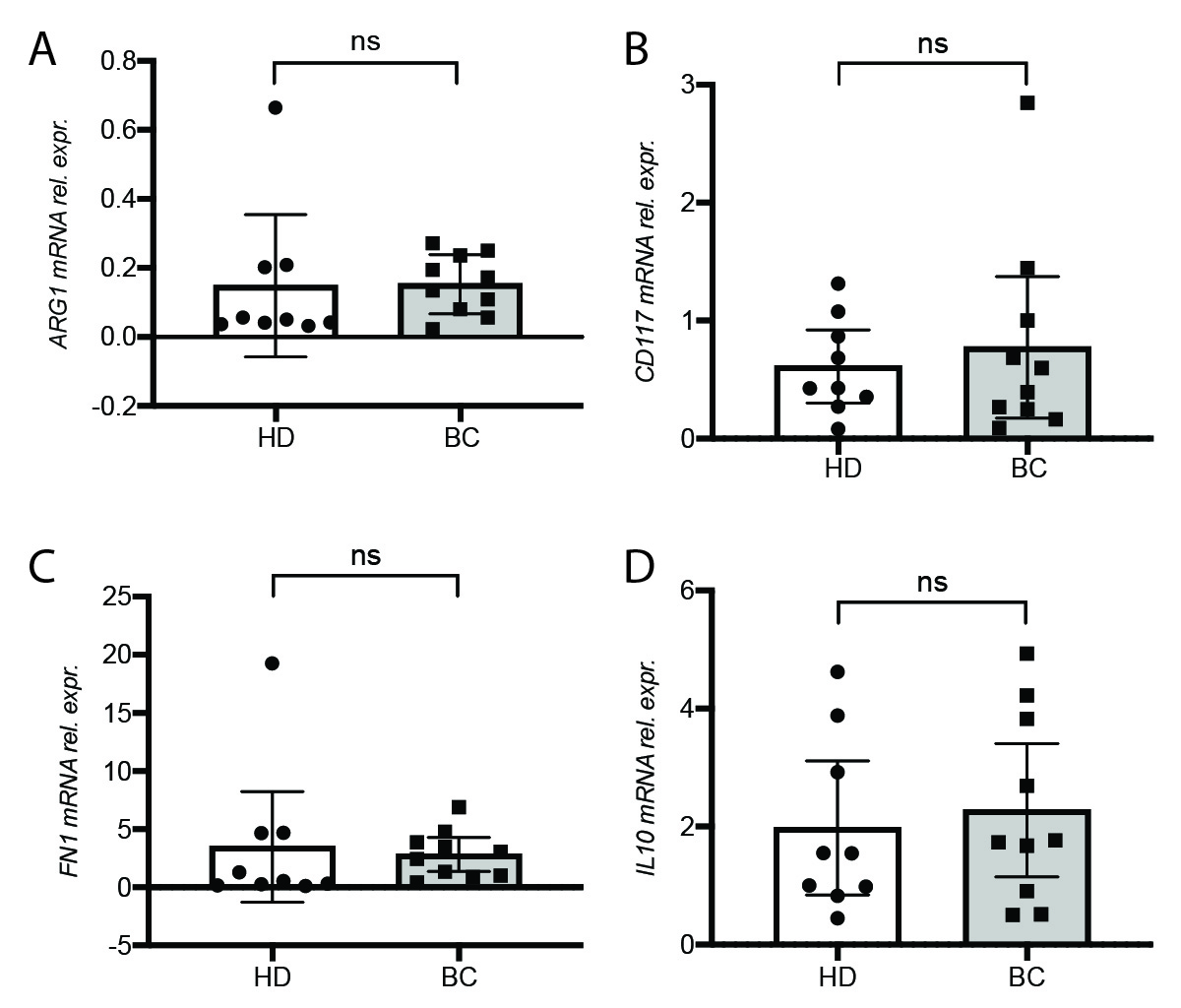


Supplementary Figure S5. No significant differences observed in mRNA levels of CD117 and M2 polarization markers in blood leukocytes of breast cancer patients vs healthy donors.

Relative expression levels of **(A)** Arginase 1 (ARG1), **(B)** CD117, **(C)** Fibronectin (FN1) and **(D)** Il-10 mRNA in the leukocytes of breast cancer patients (BC) at time of first diagnosis and age matched healthy donors (HD). Results are represented as mean +/- SD. Ns, nonsignificant.


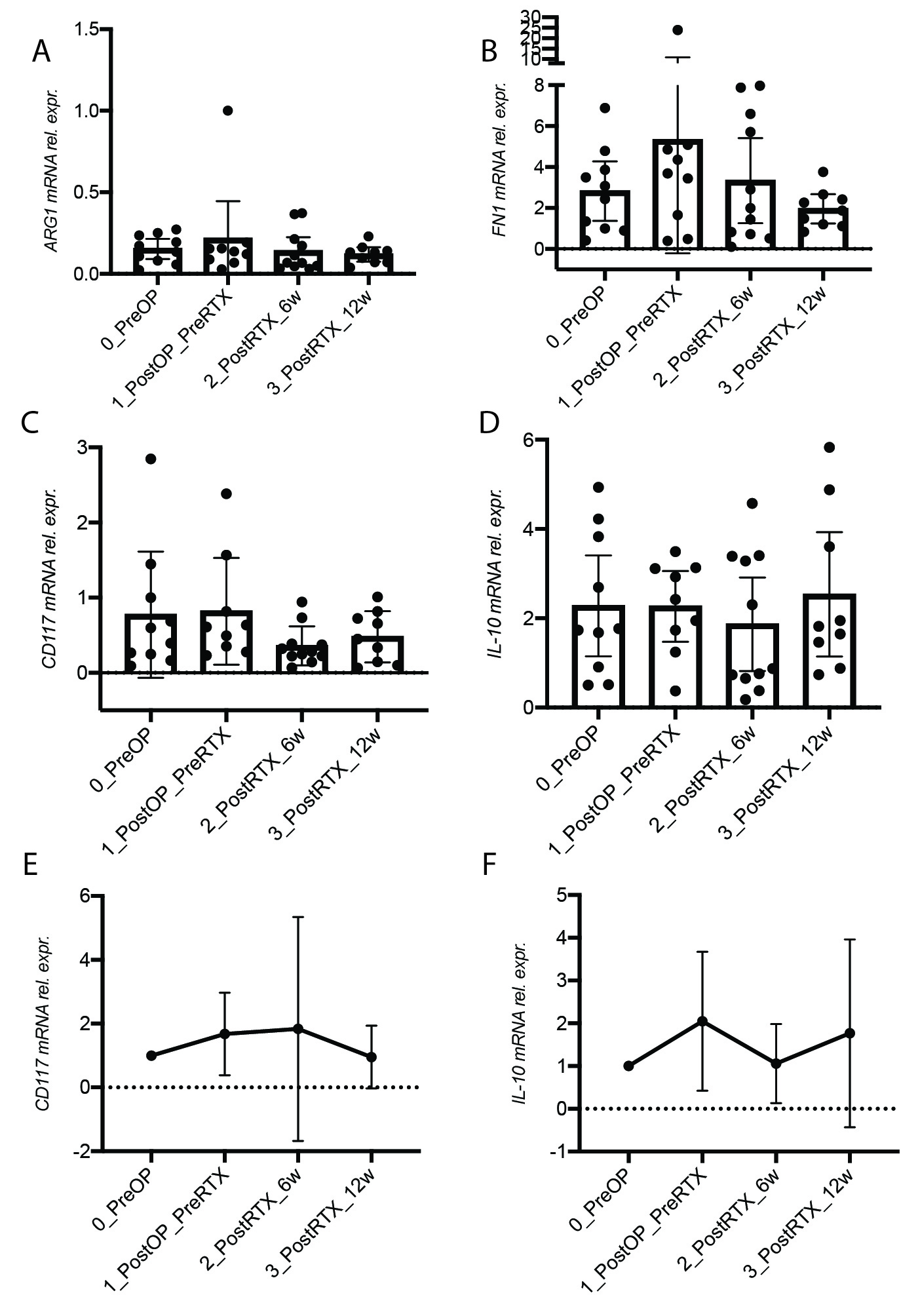


Supplementary Figure S6. No significant differences are observed in mRNA level of CD117 and M2 polarization markers in blood leukocytes of patients during radiotherapy.

Relative expression levels **(A)** Arginase 1 (ARG1), **(B)** Fibronectin (FN1), **(C)** CD117, **(D)** IL-10 in total circulating leukocytes of breast cancer patients at the indicated time-points during therapy. Relative mRNA expression to 0_PreOP for **(E)** CD117 and **(F)** IL-10 over indicated time. Cell analysis and quantification were performed by flow cytometry with FlowJo software and results are represented as mean values +/- SD.
